# Cooperativity: a competition of definitions

**DOI:** 10.1007/s00285-016-1069-2

**Published:** 2016-10-26

**Authors:** Melanie I. Stefan

**Affiliations:** Centre for Integrative Physiology, Edinburgh Medical School: Biomedical Sciences, University of Edinburgh, Edinburgh, UK

**Keywords:** Cooperative binding, Cooperativity, Biochemistry, 82, 92

## Abstract

Cooperativity as a concept is easy to grasp intuitively, but surprisingly hard to define. Two recent papers shed light on the issue and continue the debate on how best to define cooperative binding.

Cooperative ligand binding (reviewed in Stefan and Novère 2013) is a strange concept: It is relatively easy to grasp intuitively, but very hard to define exactly. Intuitively, positive cooperativity at least seems straight-forward: If some binding sites of a multivalent protein are already bound to ligand, then the other binding sites will attract ligand molecules more easily as well. This is conceptually related to effects familiar from other fields, where resources (of some kind) are allocated preferentially to those who already have some—a phenomenon often referred to as the “Matthew effect” (Merton 1968). It turns out, however, that an exact definition of cooperative binding is harder to come by. Two recent papers Abeliovich (2016), Martini et al. (2016) in the Journal of Mathematical Biology have contributed to the debate on how cooperative binding should be defined and conceptualised.

One traditional definition of cooperative binding uses the Hill coefficient (the slope of the Hill plot at half-maximal ligand saturation). The problem with this definition is that the half-maximal saturation point is somewhat arbitrarily chosen and is not necessarily representative of the overall binding curve (Abeliovich 2016; Martini et al. 2016). To address this problem, Abeliovich (2016) generalises the concept of a Hill coefficient to the entire binding curve, and thus to the entire range of possible ligand concentrations. He makes use of the fact that the slope of the Hill curve can be interpreted in terms of the variance ($\sigma ^2$) of the distribution of macro-states in the system under consideration. If the binding sites are independent, the variance is that of a binomial system ($\sigma ^2_{\text {bin}}$). Comparing this theoretical binomial variance to that of the system under consideration ($\sigma ^2_{\text {sys}}$) gives an indication of cooperativity. We can define the difference between a non-cooperative system and the system we are studying as $\varDelta = 1-\frac{\sigma ^2_{\text {sys}}}{\sigma ^2_{\text {bin}}}$ and define $I$ as the integral of $\varDelta$ over $\log [L]$, where $[L]$ is the concentration of ligand. Provided the integrand does not change sign, we get $I<0$ for systems with positive cooperativity and $I>0$ for negative cooperativity.

The definition given by Abeliovich (2016) is only one in a long history of definitions of cooperative binding. In their recent paper, Martini et al. (2016) review this history and show that the different definitions do not necessarily coincide: Given any two definitions, examples can often be found that satisfy one, but not the other. Historically, different views of cooperativity seem to be related to the different conceptualisations of ligand binding (reviewed in Stefan and Novère 2013). While some of the mathematical frameworks describing ligand binding can be translated into each other (Stefan et al. 2009), others are non-overlapping, thereby naturally giving rise to non-overlapping concepts of cooperativity. What is more, those definitions that are the most general also seem to be the least useful, in the sense that they can detect cooperativity, but not quantify it (Martini et al. 2016). In addition, the concepts of “positive” and “negative” cooperativity might be less well defined than we tend to intuitively think. For instance, a system with negative cooperativity between individual binding sites can display an overall binding profile that displays positive cooperativity (a phenomenon Martini et al. 2016 describe as “the enemy of my enemy is my friend”). Finally, cooperativity can be dependent on context, including on ligand concentration, a phenomenon that has also been pointed out by others (Edelstein et al. 2010; Ha and Ferrell 2016).

What does this mean? The concept of cooperativity seems to be more fickle and less clear-cut than most would assume. It gets even more complex when considering context phenomena such as ligand depletion (Edelstein et al. 2010; Ha and Ferrell 2016). And in all this, we have not even talked about other types of cooperativity, for instance that of ligand-dependent conformational change (Edelstein and Novère 2013). So far, there is no single definition of cooperativity that is both general enough to encompass all other definitions and useful in that it allows cooperativity—negative and positive—to be quantified. In the absence of one model of cooperativity “to rule them all”, what can we do? Martini et al. (2016) call on all of us to carefully clarify our underlying assumptions when reporting on cooperative phenomena. In addition, it is becoming increasingly clear that there are situations where the single label “cooperative” (or “positively/negatively cooperative”) does not do justice to a complex binding system. In those cases, we might have to let go of the idea of cooperativity as a global descriptor of a binding curve altogether and instead clarify the level (individual binding site or entire molecule) and molecular context in which cooperativity can be observed. Without those additional qualifiers, a global description of a biochemical system as “cooperative” does not carry enough information to be useful.
